# Walking with rollator: a systematic review of gait parameters in older persons

**DOI:** 10.1186/s11556-019-0222-5

**Published:** 2019-09-10

**Authors:** Marion Mundt, Joao Pedro Batista, Bernd Markert, Cornelius Bollheimer, Thea Laurentius

**Affiliations:** 1 Institute of General Mechanics, RWTH Aachen University, Templergraben 64, 52062 Aachen, Germany; 2 Department of Geriatrics, RWTH Aachen University Hospital, Pauwelsstraße 30, 52074 Aachen, Germany

**Keywords:** Rollator, Wheeled Walker, Walker, Four-wheeled-Walker, Assisted gait, Biomechanics, Geriatrics, Systematic review

## Abstract

**Background:**

The aging population increasingly needs assistive technologies, such as rollators, to function and live less dependently. Rollators are designed to decrease the risk of falls by improving the gait mechanics of their users. However, data on the biomechanics of rollator assisted gait of older adults are limited, or mostly derived from experiments with younger adults.

**Methods and results:**

This review summarises the data from 18 independent studies on the kinematic and kinetic gait parameters of assisted gait of older persons. All of these studies evaluated spatio-temporal parameters, but not joint angles or moments.

**Conclusion:**

Due to the limited research on rollator supported gait in older adults, the number of parameters that could be analysed in this systematic review was restricted. Further research in the analysis of spatio-temporal parameters and a higher standardisation in clinical research will be necessary.

## Background

Due to the aging society there is an increasing need for appropriate technical devices for less dependent living [1]. Walking aids, especially the so-called rollators, are frequently prescribed for older persons with gait and balance disorders to improve mobility [2]. The term “rollator”, as defined by the International Organisation for Standardisation [3], is frequently used synonymous with the term “walker”, “wheeled walker”, “four-wheeled walker”, “rolling walker” or “walking frame”. Throughout this review the term “rollator” will comprise all these synonyms. Rollators are defined as walking aids with built-in handgrips and three or more legs of which two or more are having wheels, which provide support whilst walking. Additionally, they need to be equipped with a seat for resting [3].

Falls are a major issue in older adults. About one third of the people aged older than 65 years fall once a year and half of those aged 80 years and older fall every year [4]. The risks for falls are manifold and mainly related to prior history of falls, functional impairment, use of walking aids, cognitive impairment or dementia, impaired mobility or low activity level, balance abnormalities, medications, and low muscle strength [4]. The use of a walking aid is a surrogate for poor walking performance [5–7]. Nevertheless, it seems paradox to refer walking aids to risk factors for falls, as they are supposed to increase users’ base of support and improve balance performance. Rollators are particularly prescribed to improve postural stability in patients with muscular weaknesses and balance impairments. Nevertheless, missing instructions, inappropriate, unstable usage or the design of the walking aid jeopardise the aforementioned benefits [8–10].

Some observational studies have been conducted to analyse the influence of rollator use on gait biomechanics and to get in-depth information on the immanent risk of falls. In an attempt to analyse the biomechanics of walking with a rollator, research was conducted with healthy young adults to compare unsupported versus rollator-assisted gait [11]. However, the extrapolation of the results from young people to older persons is limited as gait parameters of healthy older people differ from those of young people [12–15].

The aim of this review is to investigate the influence of a rollator on the walking biomechanics in older persons and to gain more insight into the gait parameters evaluated during rollator supported gait. We hypothesise that the biomechanics – kinematic, kinetic and spatio-temporal features – of rollator supported gait differ from unsupported gait in older persons. The further understanding of rollator supported gait is especially relevant due to the increasing development of so called ‘smart’ rollators [16–19].

## Methods

### Study selection

A systematic literature search was conducted to find related works to the hypothesis stated. The review process was divided into four phases as shown in.

**Fig. 1:**
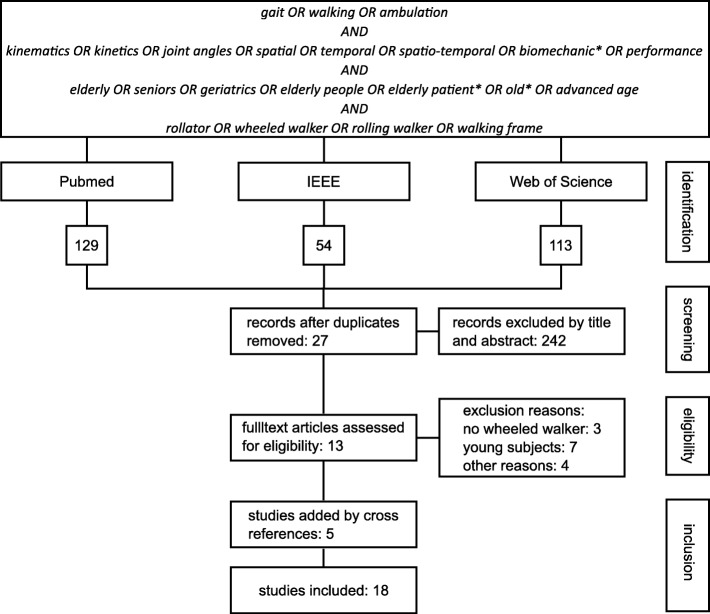
Scheme of the literature search

Figure 1 An automated search in the main databases, namely, PubMed/MEDLINE, IEEE and Web of Science was undertaken to identify relevant publications. The search terms were defined as (gait OR walking OR ambulation) AND (kinematics OR kinetics OR joint angles OR spatial OR temporal OR spatio-temporal OR biomechanic* OR performance) AND (elderly OR seniors OR geriatrics OR elderly people OR elderly patient* OR old* OR advanced age) AND (rollator OR wheeled walker OR rolling walker OR walking frame). Only publications in English language were considered. The publication period investigated was from the beginning of each database until June 2019. Inclusion criteria were defined as: (1) evaluation of kinematic gait parameters – joint angles, gait phases or spatio-temporal features – or kinetic gait parameters – ground reaction force or joint moments – of rollator supported gait and (2) participants aged 65 years or older. Any type of rollator that met the definition of a rollator [3] was included. Studies including both a rollator and additionally other assistive devices were also included to assess differences between the devices

In the second phase, titles and abstracts were screened and publications, which did not meet the aforementioned criteria, were excluded. In the third phase, the full texts of the remaining publications were assessed and those that were ineligible, for not covering the set criteria, were excluded. In the fourth phase, all remaining publications were evaluated and the references checked for further publications, which could be included in this review.

### Types of studies

This review was based on scientific journal papers and conference proceedings analysing gait biomechanics of older rollator users. Book chapters and review papers were excluded.

### Data extraction

Two independent researchers performed the data extraction and the results were compared afterwards. Disagreements were discussed and solved. In exceptional cases where they disagreed, a third researcher was consulted. Considering the methodological quality of the studies, the two reviewers focused on the following topics: number of participants; study description; type of measurement system; type of walking aid; gait parameters evaluated.

### Quality assessment

Due to the inhomogeneous objectives of the studies included, the quality assessment of the different studies was performed using an adapted questionnaire based on the Critical Appraisal Skills Program (CASP) specifically developed for this review. The questionnaire comprised five questions that could be answered by: “Yes”, “No” and “Partly”, in case there was not enough information. These three answers were scored as: “Yes” = 1.0, “Partly” = 0.5, and “No” = 0.0. As this review combines studies related to medicine and engineering, the quality assessment tool was not used as an exclusion criterion, but as an instrument to objectively compare different publications. The questionnaire used is presented in Table 1.

**Table 1:** Quality assessment of studies

No.	Question
Q1	Are the aims of the research clearly defined?
Q2	Is the tested population clearly described?
Q3	Were the methods for performing the test described in sufficient detail?
Q4	Are the findings of the study clearly stated and results reported?
Q5	Are the limitations of the study analysed explicitly?

## Results

This section was divided into two parts. The first subsection deals with the results from the quality assessment. The second subsection provides a detailed study description summarising the tested population, gait parameters evaluated, measurement systems used and results of the different studies.

The literature search yielded 18 papers that met the inclusion criteria (cf Fig. 1).

### Quality assessment

The results of the quality assessment show that most included studies were of high methodological quality according to the assessment tool used. Five studies fully scored in the questionnaire. Seven studies failed in the analysis of possible limitations. Only one study failed completely in the description of the participants (cf. Table 2).

**Table 2:** Results of the quality assessment questionnaire

	Q1	Q2	Q3	Q4	Q5
Costamagna et al. [5]	1	0.5	1	1	1
Choi et al. [20]	1	1	1	1	1
Härdi et al. [21]	1	1	1	1	1
Hunter et al. [22]	1	1	0.5	1	1
Kegelmeyer et al. [23]	1	1	0.5	1	0
Lindemann et al. [7]	1	0.5	0.5	1	1
Lindemann et al. [6]	1	0.5	1	1	1
Liu et al. [24]	0.5	1	0.5	1	0
Mahoney et al. [25]	1	1	1	1	0
Martins et al. [26]	1	0.5	0.5	1	0
Protas et al. [27]	1	0.5	1	1	1
Rampp et al. [28]	1	0.5	1	0.5	0
Schülein et al. [2]	1	1	1	1	1
Schwenk et al. [29]	1	1	1	1	1
Tereso et al. [30]	1	0.5	1	0.5	0
Tung et al. [31]	1	1	1	1	1
Wang et al. [17]	0.5	0	0.5	0.5	0

### Study description

All studies included in this review evaluated spatio-temporal parameters or stability, but none of the studies dealt with the determination of kinetic parameters or joint angles.

#### Participants

An overview of the studies’ participants is given in Table 3. In four studies older patients with specific diseases, viz. knee osteoarthritis, Alzheimer disease or Parkinson’s disease, were analysed [22, 23, 26, 30].

**Table 3:** Overview of the studies’ participants

	No. of participants	Age (years)	Description
Costamagna et al. [5]	10 (80% f)	84.2 ± 5	geriatric inpatients
Choi et al. [20]	20 (100% f)	77.9 ± 5.9	no surgery, no pain
Härdi et al. [21]	12 (75% f)	84.3 ± 3.9	geriatric inpatients
Hunter et al. [22]	20 (50% f)	79.1 ± 7.1	Alzheimer disease patients
	22 (74% f)	68.5 ± 10.7	healthy older adults
Kegelmeyer et al. [23]	27 (22.7% f)	69.7 ± 1.3	Parkinson patients
Lindemann et al. [7]	22 (50% f)	82 (73–90)	geriatric inpatients
Lindemann et al. [6]	20 (70% f)	84.5 (75–95)	geriatric inpatients
Liu et al. [24]	33 (84.9% f)	83.5	residence of an assisted living facility
Mahoney et al. [25]	15 (6.7% f)	82.3 (70–95)	geriatric in- and outpatients
Martins et al. [26]	13 (61.5% f)	67.3 ± 0.5	knee osteoarthritis and subjected to TKA
Protas et al. [27]	10 (80% f)	73.9 ± 3.9	community-dwelling older persons
Rampp et al. [28]	116 (54.7% f)	82.1 ± 6.4	geriatric inpatients
Schülein et al. [2]	106 (56.6% f)	81.7 ± 6.2	geriatric inpatients
Schwenk et al. [29]	109 (85.3% f)	83.1 ± 5.5	geriatric inpatients
Tereso et al. [30]	7 (57.1% f)	67.3 ± 5.1	knee osteoarthritis and subjected to TKA
Tung et al. [31]	20 (50% f)	89.1 ± 4.0	residence of an assisted living facility,
		83.1 ± 3.2	community-dwelling older persons
Wang et al. [17]	23/25	25–65	young adults
	12/25	> 69	
			older persons

#### Instrumentation

Nine studies used the GAITRite® measurement system [2, 7, 21, 23, 24, 27–29, 31]. In two studies an inertial sensor (Shimmer 2R) was additionally placed on the foot to collect data [2, 28]. Only two papers used a 3D motion analysis system with eight infrared cameras (Eagle 4, Motion Analysis, Santa Rosa, CA, USA [20]; Vicon, Oxford, UK [5]). One paper, due to its age, used strips of inked moleskin on a paper walkway [25] to determine spatio-temporal parameters. Two studies used tri-axial accelerometers (SMI, MP6000, InvenSense), either placed on the lower back at level L4 [26] or on the trunk and foot [30], another study used different accelerometers (Locomotion Evaluation and Gait System, LEGSys™, BioSensics, Cambridge, MA) placed on the lower limbs [22], whole inertial sensors (OPAL IMUs, APDM, Portland, USA), fixed with a belt or elastic straps at the lower back (L4–5) and frontal to the left and right ankle joints [6] or inertial sensors placed on the rollator used [17]. In one study, the rollator was instrumented with four single axis load cells (Futek LCM300, FUTEK Advanced Sensor Technology Inc., Irvine, California) and corresponding transmitters (Mantracourt T24-ACMi, Mantracourt Electronics Ltd., Exeter, UK). Additionally, a pressure-sensing insole system (Medilogic insole, T&T Medilogic Medizintechnik GmbH, Schönefeld, Germany) was used [7].

The studies included in this review were inhomogeneous regarding their research aims and assistive devices used. An overview of the devices used – based on the description within the original research papers – is given in Table 4.

**Table 4:** Assistive devices used in the different studies

	Walking aids (model, manufacturer)
Costamagna et al. [5]	instrumented four-wheeled walker
Choi et al. [20]	four-wheeled walker (V4208, Jinsan Medical, Seoul, Korea)
Härdi et al. [21]	single-tip cane
	forearm crutch
	four-wheeled walker
Hunter et al. [22]	four-wheeled walker
Kegelmeyer et al. [23]	aluminium straight cane (Harvey Surgical Supply Corporation)
	standard walker (Graham-Field Health Products)
	two-wheeled walker with fixed wheels (Medline Industries)
	four-wheeled walker with front swivel casters (Invacare Corporation)
	U-Step walker with six swivel wheels and a laser (In-Step Mobility Products)
Lindemann et al. [7]	four-wheeled walker, of which the front wheels were 360° rotatable for navigation and the rear wheels were fixed
Lindemann et al. [6]	four-wheeled walker (Ideal, Meyra, Kalletal-Kalldorf, Germany) of which the 2 front wheels were 360° rotatable
Liu et al. [24]	rolling walker
Mahoney et al. [25]	two-wheeled walker (Lumex Incorporated, model number 6054)
	three-wheeled walker (Rajowalt Corporation, model number 4200428)
Martins et al. [26]	crutches
	standard walker
	rollator with forearm supports (RFS ASBGo)
Protas et al. [27]	WalkAbout
	standard wheeled walker
	Merry Walker
Rampp et al. [28]	four-wheeled walker (Bischoff and Bischoff GmbH, Model B)
Schülein et al. [2]	four-wheeled walker (Bischoff and Bischoff GmbH, Model B)
Schwenk et al. [29]	four-wheeled walker
Tereso et al. [30]	crutches
	standard walker
	rollator with forearm supports (RFS)
Tung et al. [31]	rollator
Wang et al. [17]	instrumented three-wheeled walker

#### Parameters

One study analysed the influence of different balance abilities and handgrip heights on spatio-temporal gait parameters. Unfortunately, they did not compare the results to unsupported gait [20]. The results of this study were contradictory for both investigated groups – good and bad balance capabilities – therefore a distinct conclusion cannot be drawn to evaluate the influence of the handgrip height.

Another study analysed the influence of a rollator in Alzheimer disease patients and a healthy control group [22]. They found statistically significant differences between the two groups regarding the gait velocity and stride time variability. Additionally, they showed that the use of a rollator increased the cognitive demands compared to unassisted walking. This resulted in a decrease in gait velocity that was greater in adults with Alzheimer disease than in the healthy control group.

The following part of this section was subdivided into four parts. In the first part, the results of spatio-temporal gait parameter measurement of studies comparing frequent rollator users (FUs) and first time users (FTUs) were summarised. In the second part, the outcomes of spatio-temporal gait parameters in the same population with and without the use of a rollator are presented. Although the absolute values and the magnitude of changes between FUs and FTUs differed, the trend towards improvement appeared similar [2]. Therefore, all papers comparing either FUs or FTUs with and without the support of a rollator were combined. In the third part, rollator supported gait was compared to the gait using other assistive devices, e.g. crutches, canes, walkers and two-wheeled walkers. In the fourth part, studies analysing rollator support during different tasks of daily living were summarised.

##### Comparison of frequent rollator users to first time rollator users

Three studies [2, 24, 31] compared spatio-temporal parameters between frequent rollator users (FUs) and first time rollator users (FTUs). Only the parameter “gait velocity” was measured by all three studies, showing either no difference [31] or a higher velocity for FTUs [2, 24]. Additionally, an increase in stride length was found in two studies for FTUs [2, 24] (cf. Table 5).

**Table 5:** Gait parameters showing significant differences between first time rollator users (FUs) and frequent rollator users (FTUs)

	gait velocity	cadence	swing time	stance time	double support time	stride/step length	stride time variability	toe off angle	heel strike angle	max. Toe clearance	step width
Liu et al. [24]	+	+	+	–	–	+					
Schülein et al. [2]	+					+	–	–	–	–	
Tung et al. [31]	o	o									o

+ higher in FTUs; − lower in FTUs; o no significant difference (*p* < 0.05)

##### Comparison of gait parameters with and without the support of a rollator

Eight studies analysed the influence of the use of a rollator on the spatio-temporal gait parameters. Two studies compared the gait parameters of FUs only [2, 21], two studies combined FUs and FTUs to one group [28, 29] and five studies analysed FTUs only [2, 23–25, 27]. All studies investigating FUs revealed an increase in gait velocity, swing time and stride length when using a rollator, while these changes could not be found in FTUs. There were no other parameters analysed across these studies (cf. Table 6).

**Table 6:** Results of the comparison of the spatio-temporal parameters of FUs and FTUs with and without rollator

	gait velocity	stride time	step time	swing time	stance time	double support time	cadence	stride/step length	step width	base of support	toe off angle	heel strike angle	max. Toe clearance
Härdi et al.[21]^a^	+	o				–	o	+		–			
Schülein et al.[2]^a^	+			+				+			+	+	–
Rampp et al.[28]^b^		o		+	–			+					
Schwenk et al.[29]^b^	+	–				–	+	+		–			
Kegelmeyer et al. [23]^c^	o	o	o	o		o							
Liu et al.[24]^c^	–				+	+	–	–					
Mahoney et al.[25]^c^								o	–				
Protas et al. [27]^c^	o						o	o					
Schülein et al.[2]^c^	+			+				+			+	+	–

^a^FUs; ^b^ FUs and FTUs; ^c^ FTUs; + increase with the use of a rollator; − decrease with the use of a rollator; o no significant difference

##### Comparison of gait parameters using different assistive devices

In five studies other assistive devices than rollators were used [21, 23, 25, 26, 30]. The comparison of a cane and a rollator showed that the use of a cane and a rollator reveals gait patterns much like those without a walking aid, but the gait velocity was significantly decreased using a cane (rollator: 1.01 ± 0.04 ms^− 1^, cane: 0.94 ± 0.05 ms^− 1^) [23]. Both assistive devices led to an improved stride length and, in general, higher stability (improved stance and swing relationship, double support, cadence) [21, 23]. The use of a single crutch led to the same results as using a cane [21].

The use of two crutches led to an unsymmetrical gait whereas the use of a rollator maintained the symmetry patterns of the stride length, step length, stance and swing duration, leg speed, double support duration and step time of natural gait [26]. Additionally, crutches caused a longer stride time and a longer stance phase compared to the percentage in swing. While crutches caused a shift in stance-swing percentage to 67.7–32.4%, this ratio was 57.7–42.3% when using a rollator [30].

Walkers without wheels induced asymmetric gait patterns similar to those of crutches, because they have to be moved before each step [26]. Thereby, the gait velocity and the stride length were decreased [23]. They also extended the stride time and particularly influence the stance-swing ratio (67.6–32.4%) [30].

The two-wheeled walker also needed to be lifted before moving which led to a lower gait velocity and shorter stride length [23, 25]. The stride width was not affected by using this device [25].

##### Comparison of different tasks of daily living

There were different studies of one research group analysing the use of a rollator during different tasks of daily living [5–7] and an additional one analysing not only straight walking but also turning [17].

Costamagna et al. [5] used an instrumented rollator to analyse the stability margin, which is a direct measure for the closeness to tipping. Therefore, they analysed the foot placement in relation to the rollator and the device loading during 5 m straight line walking, 90° and 180° turning; obstacle crossing; 2.5 m backwards walking and negotiating a 50 mm step up at self-selected speed. They found the stability in straight walking to be higher than in all other tasks and no difference between those. An increase in stability was found with an increase in device loading. Nevertheless, a larger stability did not necessarily correlate with safer gait and lower risk of falls because a differentiation between users using the rollator as balance aid and users transferring weight on the device cannot be found when analysing the absolute SM results. Therefore, the analysis of normalised values needs to be undertaken to draw inter-participants’ conclusions. Anyway, the stability within one person between different tasks can be compared.

Lindemann et al. analysed gait speed, stride length, cadence and walk-ratio (step length/cadence) during different inclines with and without using a rollator [6]. During uphill walking with the rollator, the gait speed was slower compared to level walking (0.79 m/s vs. 1.07 m/s) and the walk-ratio was slightly worse (0.54 m/(steps/min) vs. 0.58 m/(steps/min)) because of a larger decrease in stride length (1.01 m vs. 1.25 m) than in cadence (94 steps/min versus 108 steps/min). The decrease in gait speed was smaller (17%) without a rollator than with a rollator (26%) and no change in walk-ratio without a rollator. During downhill walking with the rollator, there was no change in gait speed compared to level walking but a slightly decreased stride length (1.19 m vs. 1.25 m) and increased cadence (111 steps/min vs. 108 steps/min) causing a worse walk-ratio (0.55 m/(steps/min) vs. 0.58 m/(steps/min)). The decrease in gait speed and walk-ratio was smaller (4, 4%) without a rollator than with a rollator (8, 5%). The walking pattern also changed on level surfaces when using a rollator: there was an increase in walk-ratio, a decrease in cadence and gait speed but no change in stride length.

In another study, Lindemann et al. analysed one common problem in rollator use: walking through a door [7]. They evaluated the time to perform the task as well as the number of interferences between the rollator and the door. The time to complete the task was shorter without a rollator (8.71 s) than with a rollator (12.86 s). In 93% there were interferences between the rollator and the door. Additionally, the gait parameters gait speed, step width and walk-ratio were analysed in a separate experiment. The gait speed was higher when walking forwards than when walking backwards with the rollator. The step width was smaller and the walk-ratio higher. The analysis of backwards walking with and without a rollator showed an increase in gait speed, a decrease in step width and a higher walk-ratio with the support of a rollator.

Wang et al. analysed straight walking as well as turns of 90° and 180° using an instrumented walker in younger and older adults [17]. They did not find any difference in gait velocity, step period, step length and gait variability between the two groups during straight walking. Only the acceleration was larger in older adults than in younger adults. During turning, the older adults needed more time to complete the turn than the younger group. Additionally, older adults needed a larger space to complete a turn. All subjects exhibited a larger acceleration during turning phases than during walking phases. This increase was smaller for older adults than for younger adults, which might be caused by the higher acceleration older adults experience during straight walking.

## Discussion

In this paper, we aimed to review systematically those studies analysing biomechanical gait parameters of rollator supported gait in persons aged 65 years and older with the underlying hypothesis whether the rollator supported gait differs from unsupported gait in older persons. Regarding the spatio-temporal parameters, the hypothesis could be accepted.

The studies included in this review were very heterogeneous regarding their research question, assistive devices and study design. Therefore, the quality assessment results were only used to guide the interpretation of review findings and to indicate the strength of inferences. Three studies had different objectives than that proposed for this review: one covers the validation of a new measurement system [28], another the analysis of a new assistive device [27], and the third one analysed machine learning approaches [26]. All the other studies aimed to present further understanding of clinically relevant parameters to improve the risk of falls. Although the studies covered different research topics, all of them showed high scores in the quality assessment questionnaire, indicating an overall high quality of studies that investigate older adults.

The number of studies analysing rollator supported gait conducted with older persons was restricted and did not consider the investigation of joint angles or joint moments. Therefore, mainly spatio-temporal parameters could be considered in this study. In some cases, the studies included in this review reveal contradictory findings (cf. Tables 5 and 6). These differences could be caused by the inhomogeneous age of the participants of the single studies (cf. Table 3). Additionally, the disease status of the participants was not clearly defined in some studies (cf. Table 2) and the sample size was in generally small (cf. Table 3).

The use of a rollator caused higher cognitive demands than free walking [22]. This might cause, that the use of a rollator had a positive impact on the gait velocity, swing time and stride length of FUs, but these parameters did not change in FTUs (cf. Table 6). This might indicate that it is necessary to learn the correct use – regarding both the adjustment and handling – of a rollator to benefit from it. Another possible explanation might be, that the change in these parameters can be used as a quantitative measure for recommending the use of a walking aid. The comparison of different assistive devices revealed that a rollator allows the users to keep more natural gait patterns than canes, crutches, walkers and two-wheeled walkers that led to a decreased gait symmetry [21, 23, 25, 26, 30].

The quality of gait was analysed in few studies only. First time users showed a worse quality of gait (cf. Table 5), which also supports the finding that it is necessary to train the use of a rollator. The comparison of free walking and rollator supported walking reveals contradictory findings regarding the gait quality (cf. Table 6). Further research on parameters with respect to variability, balance, symmetry, and foot movement will be necessary.

The analysis of activities of daily living highlighted the necessity of training these activities with the rollator user in detail to avoid difficulties and an increased risk of falls [5–7, 17]. Tung et al. [19] analysed three neurological intensive rehabilitation in-patients who used rollators to address balance impairments, during a laboratory and ambulation assessment. The participants had to execute different activities of daily living with an instrumented rollator. The results reveal the necessity of not only studying gait in laboratory conditions, but especially in everyday conditions. For this purpose, Cheng et al. [32] suggested the use of wearable inertial sensors to determine the interaction between the rollator, the user and the walking environment.

Although the hypothesis of this review could be accepted regarding the spatio-temporal parameters, further research will be necessary to expand these findings to the analysis of joint angles and moments to get further insight in the gait mechanics of rollator assisted gait.

## Conclusion

Due to the limited research on rollator supported gait in older persons, the number of parameters that could be analysed in this systematic review were restricted. Further research in the analysis of spatio-temporal parameters and a higher standardisation in clinical research will be necessary. Additionally, there were no information in the literature regarding the joint kinematics or kinetics of rollator supported gait in older persons. Although the use of a rollator may be the best option to walk safely, the high rate of falls in rollator users is still a major concern that still needs to be addressed. Additionally, the rollator as such needs further improvements for a better handling.

## Data Availability

Not applicable.

## Abbreviations

FTUs: First time rollator users
FUs: Frequent rollator users
